# Airway collapse hinders recovery in bronchoscopy therapy for postintubation tracheal stenosis patients

**DOI:** 10.1007/s00405-024-08602-3

**Published:** 2024-04-06

**Authors:** Mingyuan Yang, Hong Li, Yunzhi Zhou, Hao Li, Huafeng Wei, Qinghao Cheng

**Affiliations:** 1 Center of Anesthesiology and Pain, Emergency General Hospital, Beijing, 100028 China; 2 Department of Pulmonary and Critical Care Medicine, Emergency General Hospital, Beijing, China; 3 Department of Anesthesiology and Critical Care, University of Pennsylvania, Philadelphia, PA 19104 USA

**Keywords:** Postintubation tracheal stenosis, Bronchoscopy intervention, Malacia, Collapse, Stenosis, Recovery and ultimate cure, Perioperative complication

## Abstract

**Background:**

Expiratory central airway collapse (ECAC) following postintubation airway stenosis (PITS) is a rare phenomenon. The impact of airway malacia and collapse on the prognosis and the success rate of bronchoscopic interventional therapy in patients with PITS had been inadequately investigated.

**Objective:**

The aim of this research was to assess the influence of airway malacia and collapse on the efficacy of bronchoscopic interventional therapy in patients with PITS.

**Design:**

This retrospective analysis examined the medical documentation of individuals diagnosed with PITS who underwent bronchoscopic intervention at the tertiary interventional pulmonology center of Emergency General Hospital from 2014 to 2021.

**Main outcome measures:**

Data pertaining to preoperative, perioperative, and postoperative stages were documented and subjected to analysis.

**Results:**

The patients in malacia and collapse group (MC group) exhibited a higher frequency of perioperative complications, including intraoperative hypoxemia, need for reoperation within 24 h, and postoperative intensive care unit admission rate (*P* < 0.05, respectively). Meanwhile, patients in group MC demonstrated significantly worse postoperative scores (higher mMRC score and lower KPS score) compared to those in pure stenosis group (*P* < 0.05, respectively), along with higher degrees of stenosis after treatment and a lower success rate of bronchoscopic intervention therapy cured (*P* < 0.05, respectively). Pearson analysis results showed that these terms were all significantly correlated with the occurrence of airway malacia and collapse in the airway (*P* < 0.05, respectively).

**Conclusion:**

The presence of malacia or collapse in patients with PITS was associated with increased perioperative complications following bronchoscopic interventional therapy, and significantly reduced the long-term cure rate compared to patients with pure tracheal stenosis.

*Trial registration *Chinese Clinical Trial Registry on 06/12/2021. Registration number: ChiCTR2100053991.

## Background

Airway obstruction (AO) refers to the blockage of the tracheobronchial passage caused by both benign and malignant lesions. Among benign etiologies, postintubation tracheal stenosis (PITS) stands out as one of the predominant causes, exhibiting a reported incidence rate of approximately 1% [1]. The main etiology is ischemic injury to the airway wall due to high cuff pressure [2], which appears several hours after intubation with fibrotic changes in the target tissue [3, 4]. The radical management of tracheal stenosis encompasses bronchoscopic interventional therapy and open surgery, with the latter offering curative potential in specific cases while not being universally tolerated or beneficial for all patients [5]. With the development of respiratory interventional techniques, bronchoscopic intervention (BI) technology can be used for airway stenosis [6–9], with a success rate of approximately 30–60%. The success rate of bronchoscopic treatment can be affected by various factors, such as the length of stenosis [10], the severity of obstruction [11] and smoking status and stenosis location [12].

Excessive and abnormal inward movement of the large airways, occurring specifically during exhalation in the respiratory cycle, is commonly known as expiratory central airway collapse (ECAC). ECAC encompasses two distinct pathophysiologic entities: excessive dynamic airway collapse (EDAC) and tracheobronchomalacia (TBM). The occurrence of ECAC following intubation is a rare phenomenon [13], and the softening may be attributed to cartilage degradation resulting from prolonged internal compression [14].

The impact of airway malacia and collapse on the prognosis and the success rate of bronchoscopic interventional therapy in patients with benign airway stenosis had been inadequately investigated. The aim of this research was to assess the influence of airway malacia and collapse on the efficacy of bronchoscopic interventional therapy in patients with PITS.

## Methods

### Study design

This study retrospectively analyzed the characteristics of stenotic lesions in patients with PITS and assesses the impact of airway collapse on the effectiveness of bronchoscopic interventional therapy at a tertiary interventional pulmonology center in Emergency General Hospital from 2014 to 2021. The study (Ethical Committee number: K21-39) received ethical approval from the Ethical Committee of Emergency General Hospital, Beijing, China (Chairperson Prof Qingyu Zeng) on 29 November 2021. Its registration date in the Chinese Clinical Trial Registry was 06/12/2021 (Registration number: ChiCTR2100053991).

### Patients

The patients were derived from the interventional pulmonology center, Emergency General Hospital, undergoing bronchoscopic intervention therapy under anesthesia from 2019 to 2021. Inclusion criteria were: (1) patients with a confirmed diagnosis of PITS by bronchoscopy; (2) scheduled for bronchoscopic intervention treatment; (3) followed up for at least 12 months. Exclusion criteria were: (1) chronicle of mental and neurological ailments, tranquilizers or sleep-inducing medications, and substance misuse involving alcohol; (2) previously diagnosed with bronchiectasis, chronic emphysema, or interstitial lung disease; (3) follow-up was less than 1 year or lost. All individuals receiving care at the interventional pulmonology center were duly notified about the potential utilization of their clinical data for clinical research purposes. They provided informed consent by signing the appropriate documentation upon admission and prior to any interventions. Monthly telephone-based interviews were conducted post-discharge for patients with PITS. In addition, the interviewers obtained oral consent from both patients and their family members during the telephone follow-up to collect patients’ information data.

Based on the results of chest CT and intraoperative bronchoscopy, we categorized the patients into two groups: those with tracheal stenosis combined with tracheomalacia and collapse were classified as the malacia and collapse group (Group MC), while those with tracheal stenosis without tracheomalacia and collapse were classified as the pure stenosis group (Group PS).

### Data collection

Preoperative general data, perioperative data and postoperative data were collected and analyzed. Preoperative general data included age, gender, intubation duration, interval days of onset breathless after extubation, modified British medical research council (mMRC) score [15], Karnofsky Performance Status (KPS) score [16], preoperative degree of stenosis and number of patients with irregularly shaped stenosis. Perioperative data included incidence of intraoperative hypoxemia, number of totals, flexible and rigid bronchoscopes, number of cryotherapy + balloon dilation, stent implantation, laser and ablation. Postoperative data include number of reoperation patients, reoperation rate within 24 h, number of postoperative intensive care unit (ICU) patient, and postoperative ICU admission rate, final degree of PITS, mMRC score, KPS score, and success rate of bronchoscopic intervention therapy cured, incidence of tracheotomy and surgical end-to-end tracheal anastomosis. Onset breathless after extubation was defined as an mMRC score of 4, indicating the patient’s inability to leave home due to severe dyspnea or experiencing dyspnea while dressing and undressing. The degree of airway stenosis evaluated the decrease in cross-sectional area by preoperative and postoperative computer tomography (CT) scan [17]. The regular shaped tracheal stenosis was characterized as either round or oval on CT, and others were defined as irregular shaped stenosis.

### Outcome

Primary outcome was the success rate of bronchoscopic intervention therapy cured. Patients were considered bronchoscopic intervention therapy cured when airway stenosis < 50% and mMRC score of ≤ 1 persisted for more than 1 year after the last BI procedure. Secondary outcomes were measures of perioperative adverse events, including incidence of intraoperative hypoxemia, reoperation rate within 24 h and postoperative ICU admission rate. The definition of intraoperative hypoxemia was when SpO_2_ during surgery fell below 90% and persisted for more than 10 s.

## Statistical analysis

Data statistical analysis was conducted using SPSS. The mean ± standard deviation format was used to present measurement data, while quantity and percentage were utilized for presenting counting data. Relevant statistical data between the two groups were compared using the Chi-square test, while the measurement data between groups were compared using univariate analysis of variance. The correlation between clinical factors and treatment condition was investigated by Pearson analysis. When the *P* value was less than 0.05, it indicated a significant statistical difference in the data.

## Results

**Fig. 1:**
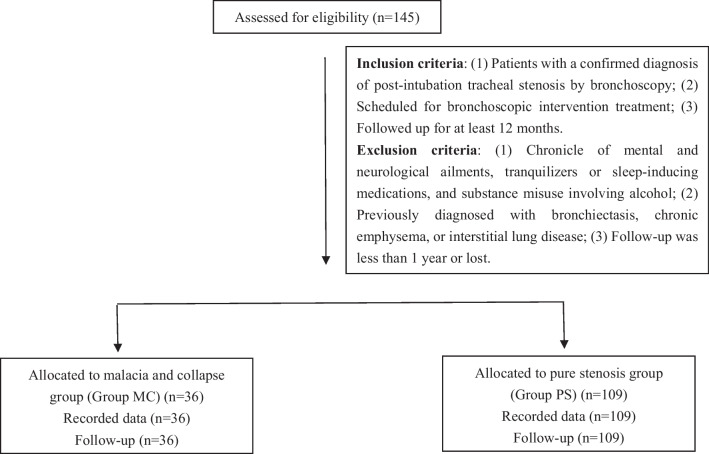
Flow diagram

145 patients were diagnosed with PITS from 2014 to 2021. They were assigned to two groups: malacia and collapse group (Group MC, *N* = 36) and pure stenosis group (Group PS, *N* = 109). The flow diagram is shown in Fig. 1.

There was no significant difference in age, gender, intubation duration, interval days of onset wheezing after extubation, mMRC score, KPS score of, and preoperative degree of stenosis between two groups (*P* > *0.05*, respectively). However, there was notable disparity in the morphological characteristics of stenosis observed between the two groups. The incidence of irregular stenosis among patients in group MC was significantly higher compared to that in group PS (*P* = 0.001). The details are shown in Table 1.

**Table 1:** Comparison of general and preoperative airway characteristics between the two groups

Group	Group MC (*n* = 36)	Group PS (*n* = 109)	*P* value
Age, years	47.81 ± 20.77	47.23 ± 17.90	0.872
Male, *n* (%)	24 (66.7%)	80 (73.4%)	0.522
Intubation duration, days	10.22 ± 7.51	10.19 ± 10.03	0.987
Time of onset of breathless, days	17.00 ± 13.68	25.46 ± 25.98	0.064
Preoperative mMRC score	3.31 ± 0.89	3.36 ± 3.69	0.933
Preoperative KPS score	58.61 ± 14.76	63.39 ± 16.34	0.121
Preoperative degree of stenosis, %	75.56 ± 15.89	75.32 ± 16.26	0.940
Irregularly shaped stenosis, *n* (%)	23 (63.9%)	35 (32.1%)^*^	**0.001**

Data were expressed as mean ± standard deviation or as numbers and percentages. ^*^ was statistically significant compared with group MC. The incidence of irregular stenosis among patients in group MC was significantly higher compared to that in group PS (*P* = 0.001)

mMRC score: modified British medical research council score; KPS score: Karnofsky Performance Status score

Group MC: malacia and collapse group; Group PS: pure stenosis group

The incidence of intraoperative hypoxemia was significantly higher in group MC compared to group PS (*P* = 0.049). The patients in group MC underwent more rigid bronchoscopic intervention therapy compared to the patients in group PS (*P* = 0.038), while there was no significant difference in total procedures and flexible bronchoscope BI (*P* = 0.396, *P* = 0.111). The rate of cryotherapy + balloon dilation was significantly lower in the MC group than in the PS group (*P* = 0.040), while the rate of tracheal stent implantation was significantly higher in the MC group compared to the PS group (*P* = 0.017). There was no significant difference observed in the rate of laser and ablation treatment between the two groups (*P* = 0.097). The details are shown in Table 2.

**Table 2:** Comparison of bronchoscopy treatment characteristics between the two groups

Group	Group MC (*n* = 36)	Group PS (*n* = 109)	*P* value
Incidence of intraoperative hypoxemia, %	0.34 ± 0.36	0.23 ± 0.27^*^	**0.049**
Number of total bronchoscopes, *n* (%)	7.58 ± 5.70	9.13 ± 10.36	0.396
Number of flexible bronchoscopes, *n* (%)	5.36 ± 4.62	7.86 ± 8.95	0.111
Number of rigid bronchoscopes, *n* (%)	2.25 ± 2.23	1.28 ± 2.45^*^	**0.038**
Cryotherapy + balloon dilation, *n* (%)	4.89 ± 4.46	8.17 ± 9.12^*^	**0.040**
Stent implantation, *n* (%)	0.50 ± 0.51	0.23 ± 0.60^*^	**0.017**
Laser and ablation, *n* (%)	2.31 ± 2.49	1.61 ± 2.03	0.097

Data were expressed as mean ± standard deviation or as numbers and percentages. ^*^ was statistically significant compared with group MC. The incidence of intraoperative hypoxemia was significantly higher in group MC compared to group PS (*P* = 0.049). The patients in group MC underwent more rigid bronchoscopic intervention therapy and stent implantation therapy compared to the patients in group PS (*P* = 0.038, *P* = 0.017), and the rate of cryotherapy + balloon dilation was significantly lower in the MC group than in the PS group (*P* = 0.040)

Group MC: malacia and collapse group; Group PS: pure stenosis group

The perioperative adverse events, degree of stenosis, postoperative scores, final bronchoscopic cure rate and incidence of tracheostomy exhibited significant disparities between the two groups. The number of patients who underwent emergency surgery within 24 h after the initial operation and the reoperation rate within 24 h were significantly higher in group MC compared to group PS (*P* = 0.002, *P* = 0.000), while there was also a significant increase in the number of postoperative patients requiring tracheal intubation upon return to ICU and the postoperative ICU admission rate when compared to group PS (*P* = 0.042, *P* = 0.048). Patients in group MC demonstrated significantly worse postoperative scores (higher mMRC score and lower KPS score) compared to those in group PS (*P* = 0.000, *P* = 0.002), along with higher degrees of stenosis after treatment, a lower success rate of bronchoscopic intervention therapy cured and higher incidence of tracheotomy (*P* = 0.000, *P* = 0.001, *P* = 0.001). The details are shown in Table 3.

**Table 3:** Comparison of perioperative adverse events, postoperative stenosis characteristics between two groups

Group	Group MC (*n* = 36)	Group PS (*n* = 109)	*P* value
Number of reoperation patients, *n* (%)	11 (30.6%)	9 (8.3%)^*^	**0.002**
Reoperation rate within 24 h, *n* (%)	0.07 ± 0.13	0.01 ± 0.05^*^	**0.000**
Number of postoperative ICU patient, *n* (%)	5 (13.9%)	4 (3.7%)^*^	**0.042**
Postoperative ICU admission rate, *n* (%)	0.03 ± 0.09	0.01 ± 0.04^*^	**0.048**
Postoperative degree of stenosis, %	18.90 ± 3.15	8.97 ± 0.86^*^	**0.000**
Postoperative mMRC score	1.33 ± 0.63	0.86 ± 0.54^*^	**0.000**
Postoperative KPS score	76.94 ± 17.21	86.79 ± 15.98^*^	**0.002**
Success rate of bronchoscopic intervention therapy cured, *n* (%)	12 (33.3%)	73 (67%)^*^	**0.001**
Tracheotomy, *n* (%)	7 (19.4%)	2 (1.8%)^*^	**0.001**
End-to-end anastomosis of trachea, *n* (%)	3 (8.3%)	8 (7.3%)	0.545

Data were expressed as mean ± standard deviation or as numbers and percentages. ^*^ was statistically significant compared with group MC. The number of patients who underwent emergency surgery within 24 h after the initial operation and the reoperation rate within 24 h were significantly higher in group MC compared to group PS (*P *= 0.002, *P *= 0.000), while there was also a significant increase in the number of postoperative patients requiring tracheal intubation upon return to ICU and the postoperative ICU admission rate when compared to group PS (*P *= 0.042, *P *= 0.048).  Patients in group MC demonstrated significantly worse postoperative scores (higher mMRC score and lower KPS score) compared to those in group PS (*P *= 0.000, *P *= 0.002), along with higher degrees of stenosis after treatment, a lower success rate of bronchoscopic intervention therapy cured and higher incidence of tracheotomy (*P *= 0.000, *P *= 0.001, *P *= 0.001)

mMRC score: modified British medical research council score; KPS score: Karnofsky Performance Status score

Group MC: malacia and collapse group; Group PS: pure stenosis group

**Fig. 2:**
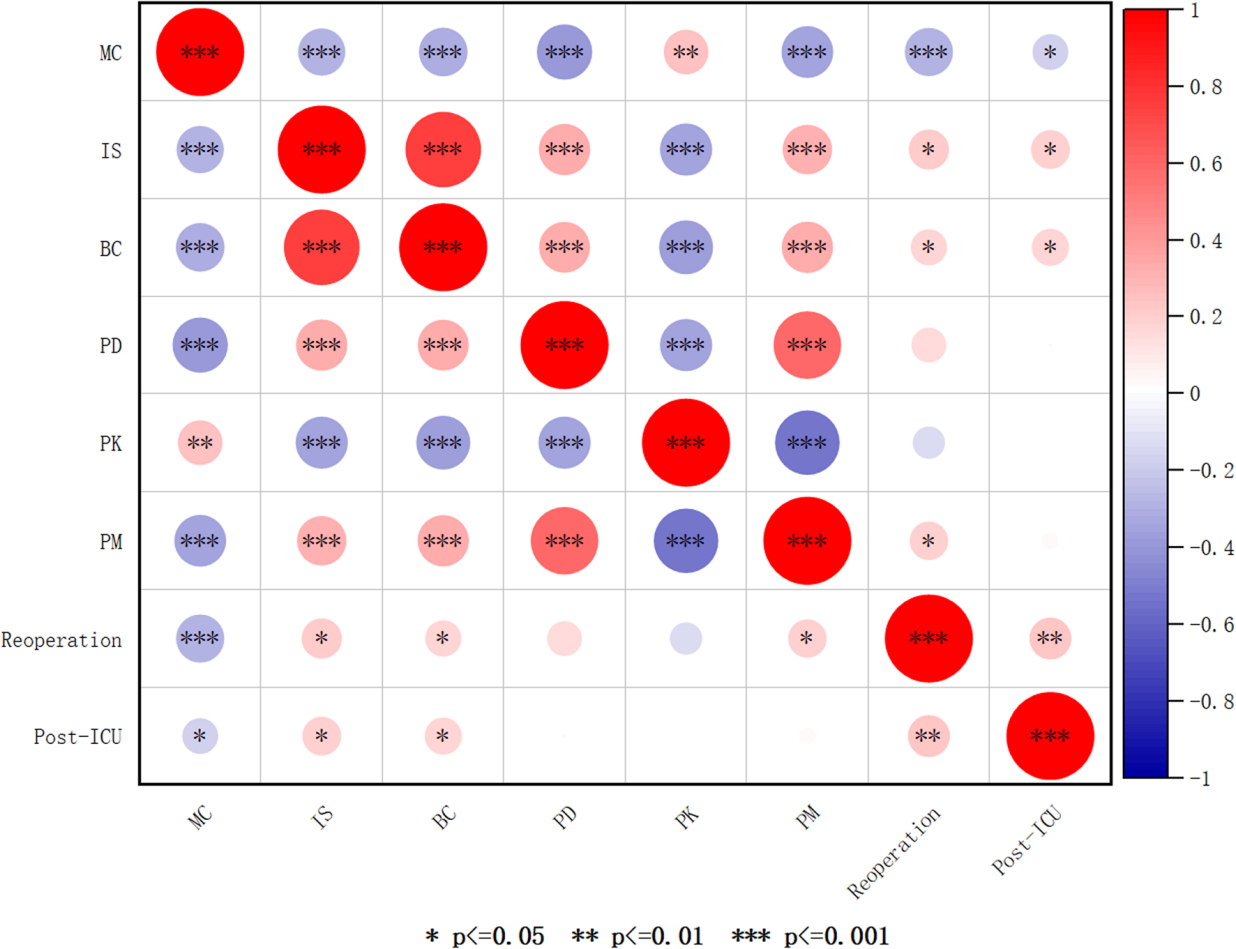
Heat map of Pearson correlation analysis of preoperative and postoperative related factors. MC: malacia and collapse; IS: irregularly shaped stenosis; BC: bronchoscopic intervention therapy cured; PD: postoperative degree of stenosis; PK: postoperative KPS score; PM: postoperative MMRC score; reoperation: reoperation rate within 24 h; post-ICU: postoperative ICU admission rate; the color red signifies a positive correlation, while the color blue indicates a negative correlation. In addition, larger circles represent higher levels of correlation, whereas smaller circles denote lower levels of correlation

Pearson analysis results showed that irregularly shaped stenosis, success rate of bronchoscopic intervention therapy cured, postoperative degree of stenosis, postoperative KPS score, postoperative mMRC score, reoperation rate within 24 h, and postoperative ICU admission rate, were all significantly correlated with the occurrence of airway malacia and collapse in the airway (*P* = 0.000,* P* = 0.000,* P* = 0.000,* P* = 0.002,* P* = 0.000, *P* = 0.000,* P* = 0.048). The details are shown in Fig. 2.

## Discussion

The incidence of airway stenosis has witnessed a significant surge once again following multiple spikes in the COVID-19 pandemic [18–20]. An increasing number of patients are experiencing PITS, particularly among those with prolonged intubation and obesity due to COVID-19 infection [21], with prevalence rates as high as 40% observed in this population [22].

With advancements in respiratory interventional techniques, the bronchoscopic intervention airway therapy has been widely employed as a primary treatment modality for tracheal stenosis [6–9, 23], exhibiting an impressive efficacy rate of up to 98.6% in patients with PITS. However, its effectiveness is notably compromised when dealing with complex cases [24]. BI can significantly improve patients’ symptoms by relieving airway stenosis [7, 25, 26], but patients with PITS often required repeated BI procedures. Bronchoscopic intervention procedures encompass a range of techniques, including laser excision, balloon dilatation, stent insertion, electrocautery, argon plasma coagulation, topical application of mitomycin C, and cryotherapy [27]. Despite the availability of various therapeutic options, patients with severe PITS, particularly those with complicated stenosis, long-standing cartilage lesions [12] or inadequate response to internal medicine bronchoscopy treatment, often necessitate consideration of tracheal reconstruction surgery, tracheotomy, or T-tube insertion [28–32]. Tracheal stenoses accompanied by malacia and inflammation are considered complex conditions, necessitating a multidisciplinary approach and often requiring surgical intervention [24].

Prolonged positioning of the endotracheal tube and excessive cuff pressure as well as repeated injury from reintubations result in irritation of the airway, heightened secretion production, and reduced blood flow [14, 33, 34]. Furthermore, repeated exposure to positive pressure ventilation has the potential to worsen airway harm through tracheal overexpansion, reduced thickness of tracheal smooth muscle, and injury to the epithelial lining [35]. These circumstances lead to the development of tracheal inflammation, either in an acute or chronic form, leading to the susceptibility of the tracheal wall. Inflammation may potentially result in airway wall weakness, thereby predisposing the trachea to collapse. The findings of previous studies indicate that approximately 1–2% of patients who underwent percutaneous tracheotomy experience the development of severe tracheomalacia [36]. TM, TBM and EDAC, often co-exist and are commonly used interchangeably in practical applications [37, 38].

Tracheomalacia can arise from the underdevelopment of tracheal cartilage during birth or the deterioration of previously healthy cartilage due to external pressure or a prolonged inflammatory reaction [39]. The tracheal wall could be compromised and its structural integrity lost due to chronic endogenous compression and the resulting inflammatory response. Tracheostomy and the presence of tracheal tubes equipped with cuffs were recognized as the predominant etiologies contributing to acquired tracheomalacia in adult patients [14, 40]. The inflammatory process is closely associated with the structural vulnerability of tracheal cartilage caused by tracheostomy and the presence of indwelling endotracheal tubes.

Balloon dilatation via flexible fiberoptic bronchoscopy is a straightforward yet efficacious approach for managing proximal benign tracheobronchial stenosis. However, its effectiveness appears to be limited in patients with airway collapse and TM. For patients suffering from severe tracheomalacia, the placement of a tracheal stent had demonstrated efficacy in alleviating symptoms. However, long-term stent placement may give rise to various associated complications [41], including granulation hyperplasia. In cases of severe granulation, timely removal of the endotracheal stent is imperative. The situation becomes more problematic when, following the removal of the endotracheal stent, patients with malacia and collapse experienced recurrent symptoms of asphyxia [42], thereby establishing a detrimental cycle that poses a threat to the patient's life.

Recurrent issues have been reported with the prolonged placement of stents, including but not limited to the accumulation of mucus, movement of the stent from its intended position, and the development of excessive tissue growth. These complications may potentially lead to airway obstruction [43, 44]. These complications may potentially lead to airway obstruction. An article reported on the abrupt respiratory decompensation observed in a patient with dynamic TBM, due to intense tissue granulation at the periphery of Y-stent that was inserted [45]. Therefore, stent placement had been often employed as a transient palliative measure for patients with tracheomalacia or as an interim intervention and bridge prior to surgery.

The lower final success rate of bronchoscopic intervention therapy in the tracheomalacia and collapse group was observed in this study, accompanied by reduced postoperative breath holding scores and limited improvement in the degree of stenosis. Interventional bronchoscopy yields satisfactory outcomes in cases of tracheal stenosis characterized by localized hypergranulation and mild to moderate airway obstruction [11]. For the complex condition of stenosis combined with malacia or collapse, the effective rate of bronchoscopic treatment is greatly reduced. Patients who have diffuse TBM may be eligible for surgical intervention if the degree of malacia exceeds that of small airway disease; this is commonly observed when their collapse affects the entire trachea uniformly and extensively [46].

Meanwhile, the procedures also exhibited a higher frequency of adverse events, especially reoperation rate within 24 h and postoperative ICU admission rate. In patients with large airway collapse, the sudden inability to ventilate can lead to life-threatening hypoxemia as a result of expiratory flow obstruction. The presence of airway collapse can often be asymptomatic, particularly in cases where the degree of airway narrowing is mild to moderate. However, as the severity of airway constriction progresses or if triggered by factors such as general anesthesia or unsuccessful mechanical ventilation withdrawal, symptoms and signs may manifest [47, 48].

Due to the presence of malacia, the shape of the tracheal stenosis lesion becomes irregular. Based on the study findings, it is evident that patients in the MC group exhibit a higher degree of morphological irregularity compared to those in the PS group. The validity of stenosis shape as another critical predictor of patient outcome has been substantiated by our previous research. The ultimate prognosis, incidence of reoperation within 24 h, and transfer back to ICU were significantly influenced by the shape of stenosis. Lesions with a more irregular shape pose greater challenges in treatment, leading to higher complication rates and worse overall prognosis. Patients with irregular stenosis had a poorer prognosis and were more likely to undergo multiple bronchoscopic treatments resulting in treatment failure, ultimately requiring surgical intervention.

The challenges faced by patients with malacia and collapse during extubation process were more severe and prone to recurrence, in addition to the risk of intraoperative hypoxemia, which distinguishes them from typical airway stenosis patients [49]. Intraoperative mortality resulting from abrupt hypoxia subsequent to airway collapse has been documented in individuals afflicted with profound obstructive alterations [50].

Rigid bronchoscopy is frequently employed as a targeted approach to alleviate the stenosis of the trachea. The utilization of rigid bronchoscopy for primary airway dilation serves as an effective means to integrate ventilation and stenosis treatment [51], circumventing the potential obstruction associated with endoscopic balloon dilation while minimizing the risk of prolonged blockage leading to asphyxiation. The management of TBM relies on the presence of expiratory central airway collapse and the effectiveness of initial airway stabilization techniques. Tracheobronchoplasty is employed as a treatment option for severe cases of TBM. Patients with tracheomalacia treated by airway bronchoscopy alone had a worse outcome than those with airway stenosis alone.

The limitations of this study are the following: (1) initially, it is important to note that this study is conducted retrospectively, potentially resulting in the exclusion or loss of certain data. Consequently, a comprehensive evaluation of the factors influencing patient prognosis may not be feasible. The implementation of prospective studies may enhance the mitigation of study bias resulting from incomplete data collection; (2) additionally, this inclusion criteria exclusively encompassed patients presenting with PITS subsequent to endotracheal intubation, thereby excluding individuals who underwent tracheotomy or cricothyroid membrane puncture. The occurrence of tracheomalacia and tracheal airway collapse following tracheotomy or cricothyroid membrane puncture presents a more intricate scenario, exhibiting slight variations compared to PITS. We will continue to explore this issue in depth in the follow-up study; (3) the complexity of tracheal stenosis extends beyond the combination of stenosis and malacia collapse. This study solely focuses on collapse, without examining factors such as stenosis shape, length, position simultaneously.

In conclusion, the success rate of bronchoscopic intervention treatment for patients with PITS combined with airway malacia and collapse is significantly lower compared to those with pure stenosis. In addition, this condition significantly impacts the occurrence of treatment complications and perioperative adverse events, particularly in PITS patients with malacia and collapse, who are more prone to experiencing a higher reoperation rate within 24 h and an increased likelihood of postoperative ICU admission. The clinical treatment of such patients requires increased attention and the selection of a more comprehensive plan based on their condition.

## Data Availability

The datasets used and/or analyzed during the current study are available from the corresponding author on reasonable request.
